# University Food Environment Assessment Methods and Their Implications: Protocol for a Systematic Review

**DOI:** 10.2196/54955

**Published:** 2024-08-23

**Authors:** Alicia Anne Dahl, Lilian Ademu, Stacy Fandetti, Ryan Harris

**Affiliations:** 1 Department of Epidemiology and Community Health University of North Carolina at Charlotte Charlotte, NC United States; 2 Texas A&M Institute for Advancing Health Through Agriculture Texas A&M AgriLife Research Centre at El Paso El Paso, TX United States; 3 J. Murrey Atkins Library University of North Carolina at Charlotte Charlotte, NC United States

**Keywords:** universities, nutrition assessment, nutrition policy, health behavior, young adult, food environment, retail food, university food environment, university food, dietary choice, food assessment, tool, university, lifestyle habit, diet, obesity, United States, weight gain

## Abstract

**Background:**

While the retail food environment has been well studied, research surrounding the university food environment is still emerging. Existing research suggests that university food environments can influence behavioral outcomes such as students’ dietary choices, which may be maintained long-term. Despite a growing interest in assessing university food environments, there is no standardized tool for completing this task. How researchers define “healthy” when assessing university food environments needs to be clarified. This paper describes the protocol for systematically reviewing literature involving university food environment assessments.

**Objective:**

This paper aimed to describe the protocol for a systematic review of the assessments of university food environments. The review will summarize previously used tools or methods and their implications.

**Methods:**

Electronic databases, including PubMed (NLM), Cochrane Library (Wiley), Web of Science (Clarivate), APA PsycINFO (EBSCO), CINAHL (Cumulative Index to Nursing & Allied Health) Complete (EBSCO), ProQuest Nursing and Allied Health, and Google Scholar were searched for papers published between 2012 and 2022 using combinations of related medical subject headings terms and keywords. The electronic databases were supplemented by reviewing the reference list for all included papers and systematic reviews returned with our search results. The review will include all study types, including randomized controlled trials, observational studies, and other pre-post designs. Papers that examine at least 1 aspect of the university food environment, such as cafeterias, campus convenience stores, and vending machines, were considered for inclusion. A total of 2 reviewers will independently screen titles and abstracts, complete a full-text review, extract data, and perform a quality assessment of included papers, with a third reviewer resolving any conflicts. The Quality Assessment for Diverse Studies (QuADS) tool was used to determine the methodological quality of selected studies. A narrative and tabular summary of the findings were presented. There will not be a meta-analysis due to the methodological heterogeneity of the included papers.

**Results:**

The initial queries of 4502 records have been executed, and papers have been screened for inclusion. Data extractions were completed in December 2023. The results of the review were accepted for publication in May 2024. The systematic review generated from this protocol will offer evidence for using different assessment tools to examine the campus food environment.

**Conclusions:**

This systematic review will summarize the tools and methods used to assess university food environments where many emerging adults spend a significant part of their young adult lives. The findings will highlight variations in practice and how “healthy” has been defined globally. This review will provide an understanding of this unique organizational food environment with implications for practice and policy.

**Trial Registration:**

PROSPERO CRD42023398073; https://www.crd.york.ac.uk/prospero/display_record.php?RecordID=398073

**International Registered Report Identifier (IRRID):**

DERR1-10.2196/54955

## Introduction

### Rationale

An optimal time to encourage healthy lifestyle habits is during young adulthood, specifically between the ages of 18 and 25 years [1-4]. This transitional period from adolescence to young adulthood is critical for developing self-identity, self-efficacy, and long-term behaviors and lifestyles [4]. Research suggests diet quality, activity patterns, and overall health decline during this transition with exposure to unfamiliar environments and routines [5-7]. For many emerging adults, this period also means transitioning to college and shifting interpersonal influences [8].

The obesity rate among young adults in the United States has risen in the past decade from 24% to 29% [9]. This increase is partially due to unhealthy dietary and physical activity behaviors [10-12], which are modifiable. Since college students make up a considerable proportion of the emerging adult population globally, university campuses may be an optimal intervention point. For instance, 38% of emerging adults in the United States attended college in 2021 [13]. Many studies suggest that the transition to college is associated with poor dietary intake and excess weight gain, especially in the first year [14-18].

Poor dietary behaviors among college students are associated with decreased health, lower academic outcomes, and lower socioeconomic outcomes [19-22]. Thus, college students are ideal for interventions designed to improve dietary choices and promote healthy lifestyles. Adopting healthy nutritional practices by young adults might persist throughout adulthood, resulting in a reduced risk of chronic diseases later in life [23,24].

The social cognitive theory emphasizes reciprocal determinism and provides a valuable framework for exploring how environmental factors or changes influence the attitudes and behaviors of college students [25]. The social cognitive theory and other related theories, such as the socio-ecological model, have been applied in several initiatives implemented within higher education in an attempt to promote healthy and environmentally sustainable campus communities [15,26,27]. Research indicates that these interventions have a significant role in shaping health behaviors among students, and college administrators play a critical part in developing and implementing policies that encourage healthy choices [26].

Beyond the design and implementation of health promotion interventions, research in this domain has examined the healthfulness of food environments [28-30], dietary and food purchasing behaviors of college students [31-34], food security among college students [35], nutrition security [36,37], food accessibility [38], menu diversity [39], and food sustainability [14,40]. These studies cut across different countries and postsecondary school contexts, providing valuable insights for designing effective nutrition policies on university campuses. An overlapping objective of most of these studies is to improve the healthfulness of campus food environments and fight the obesity epidemic among young adults.

The existing evidence regarding campus food environments and their impact on healthy eating behavior is still emerging. Recent literature suggests that university food environments offer less nutritious food options [41,42] and college students endure food and nutrition insecurity [43]. Notably, the COVID-19 pandemic altered the student experience and increased food insecurity among young adults [44]. At an institutional level, many university campuses have yet to develop policies to encourage the consumption of sustainable food options [26,45] or fail to co-ordinate efforts to improve access to safety net programs (eg, the SNAP [Supplemental Nutrition Assistance Program] program enrollment) for students experiencing food insecurity with limited resources [46]. Furthermore, opinions on improving the healthfulness of food environments, menu diversity, food security, and food accessibility are diverse and context-specific [47]. Studies on the effects of environmental changes on dietary choices provide mixed findings. For example, research on the impact of calorie posting and nutritional labeling on dietary behaviors and choices of college students suggests both significant and nonsignificant positive effects [48-52]. Despite the burgeoning body of literature on campus food environments, research on the methods used to assess campus food environments and their healthfulness is broadly lacking.

Further investigation is warranted because of the increasing interest in emerging adult health [53], campus food security, and university food environment research [54]. Universities are a unique type of organizational food environment in which they function as a “mini city” [55,56]. Many students, typically first-year students, are required to buy meal plans to obtain food on campus. As a result, these students may be limited to the options available on campus [8]. Such factors set university campuses apart from other consumer food environments and provide a unique setting for understanding its relationship with the dietary behaviors of emerging adults.

Systematic reviews of the broad retail food environment, including vending machines, exist [57-64]. In addition, researchers have conducted systematic reviews of the impact of the campus food environment or food environment interventions on students’ dietary behaviors [65-67]. However, no systematic review has investigated how universities assess their food environments, how “healthy” is defined, and the behavioral and environmental implications of conducting this type of assessment. As a result, there is a need for a concise understanding of the methods currently used to evaluate the healthfulness of university food environments to address variations in practices and understand how researchers define “healthy.”

### Objectives

This paper aimed to describe the protocol for a systematic review of the assessments of university food environments. The review will summarize previously used tools or methods and their implications. Researchers, practitioners, and policymakers can review these tools when determining how to evaluate their campus food environment in the best way. Furthermore, public health nutrition researchers and university administrators can better understand how to look at campus food environments and determine areas for improvement. The systematic review will answer the following questions: (1) How are university food environment assessments conducted? (2) How is “healthy” being defined? What guidelines are researchers using to determine “healthy?”

This systematic review would help researchers assess their campus food environments and may support the development of policies to create healthier university food environments. These actions may positively impact students’ dietary behaviors or food choices.

## Methods

### Registration and Reporting

This protocol was registered with the PROSPERO (International Prospective Register of Systematic Reviews; CRD42023398073), and was prepared following the PRISMA-P (Preferred Reporting Items for Systematic Review and Meta-Analysis Protocols) [68]. The entire systematic review will follow the PRISMA (Preferred Reporting Items for Systematic Review and Meta-Analysis) 2020 guidelines [69].

### Eligibility Criteria

#### Types of Studies

In addition to papers that focus on reporting the development or validation of measures, there are no restrictions on the type of study to allow our research team to capture assessment results that may be included as a component of behavioral or experimental studies. The review may include randomized controlled trials, observational studies, cross-sectional, nonrandomized studies, feasibility and acceptability trials, and other pre-post designs.

#### Types of Participants

The review will include studies that assess at least 1 aspect of the university food environment, including campus convenience stores, dining halls, quick-service restaurants, and vending machines. In addition, it will include studies examining the perceptions of the food environment from students, university employees, and other campus dining stakeholders.

#### Types of Interventions

This review is not solely focused on interventional studies. Instead, it will synthesize evidence from peer-reviewed, published literature on the assessments of university food environments over 10 years (between 2012 and 2022).

#### Comparators

Given the proposed focus on a descriptive summary of existing literature on university food environment assessments, studies with any comparison group and no comparison group are eligible for inclusion.

#### Types of Outcomes

The proposed review will focus only on assessment methods. Studies were included in the review if there is a tool or method detailed for how data on the campus food environment can be collected and if original research findings were included. Secondary outcomes were the guidelines or standards used to define “healthy” within these assessments (eg, federal guidelines) and any policy recommendations provided as part of the publication (eg, added sugar limits on vending machine products). Studies with no nutritional focus were excluded from the review.

#### Timing

The date range of published papers to be included in the review is from 2012 to 2022.

#### Setting

The setting includes all university, college, and campus food environments (ie, public, private, multisite, or unspecified). University medical centers may also be included. The countries of origin were unrestricted.

#### Language

We will include papers written in the English language.

### Exclusion Criteria

Regarding the criteria listed above, papers may be excluded from the review if they do not describe an assessment of the food environment. Dissertations, theses, and conference abstracts without an associated peer-reviewed publication will also be excluded from the final review.

### Information Sources

We will search the databases, such as PubMed (NLM), Cochrane Library (Wiley), Web of Science (Clarivate), APA PsycINFO (EBSCO), CINAHL (Cumulative Index to Nursing & Allied Health) Complete (EBSCO), ProQuest Nursing, and Allied Health. We will not have any restrictions when searching the databases. The electronic database searches were supplemented by reviewing the reference list for all included papers and systematic reviews returned with our search results. We will also search the gray literature on Google Scholar by examining the first 10 pages of results yielded from our search.

### Search Strategy

Both qualitative and quantitative studies were included in this systematic review search. No study design, date, or language limits were imposed on the search. At the conception of the research question, a librarian (RH) with literature-searching expertise worked to identify appropriate concepts and terminology for the research question. Our team identified 3 main areas for the search, such as food choice and eating behaviors, food environment and caloric information, and the college environment. A variety of terms were included for each concept. For food choice and environments, meal behavior, food choice, and purchasing behaviors were some of the included terms. For the concept of the food environment, a sample of included terms were meal plans, nutrition information, and food access. The college environment search included terminology such as university, college, and postsecondary. Subject term and keyword searching were applied to all searches when appropriate, depending on the database being used. The database search strategies can be found in Table S1 in Multimedia Appendix 1.

In addition, spelling variations, phrase searching, and truncation were applied to allow for a comprehensive search. The librarian (RH) conducted an initial search in November 2022, for the team to review and refine the search. All library database searches were completed in January 2023. The Google Scholar search was completed in July 2023, to capture publications that may have been indexed late for 2022.

### Data Management

Literature search results were uploaded to Covidence, an internet-based software program for systematic reviews [70]. The team used Covidence for the screening and full-text review process. At each stage, a team discussion occurred to review and revise the criteria as needed.

In total, 2 review authors (SF and LA) independently screened the titles and abstracts in Covidence to determine if they meet the inclusion criteria. A third author (AD) resolved any conflicts that arose during the screening process. Next, the papers that met the inclusion criteria were downloaded for review. Then, all reviewing authors (AD, SF, and LA) first independently reviewed 6 papers and discussed the rationale for inclusion or exclusion before moving through the full-text review process. Once the full-text review had been completed by 2 authors (SF and LA), a third author (AD) resolve any conflicts through discussion. None of the authors were blind to the journal titles, study authors, or institutions.

### Data Extraction

Based on quantitative and qualitative data, this review investigates the types of assessments used to determine the healthfulness of a university food environment. We are examining how assessments have been conducted (eg, observational and focus groups) and how “healthy” is defined or what they are using as a guide (eg, USDA [United States Department of Agriculture] guidelines and menu labeling or best practices). A total of 2 reviewers (SF and LA) extracted the information regarding the paper title, DOI (digital object identifier), author names, year, geographic location, subjects included, setting, assessment type, study design, sample size, sample type, sample characteristics, duration, study objective, primary outcome, secondary outcome, type of assessment, benchmark criteria for “healthy,” and summary of findings. A third review author (AD) reviewed the extracted data, and any adjustments or updates recommended were discussed among the reviewers. The data extraction sample tables are provided in Multimedia Appendices 2-4.

### Quality Assessment of Included Studies

The Quality Assessment for Diverse Studies (QuADS) tool was used to assess the methodological quality of selected studies (ranked on a scale of 0-3) in each of the areas, such as (1) theoretical or conceptual underpinning to the research, (2) statement of research aims, (3) clear description of the research setting and target population, (4) whether the study design is appropriate to address the stated research aims, (5) appropriate sampling to address the research aims, (6) the rationale for the choice of data collection tools, (7) whether the format and content of data collection tool is appropriate to address the stated research aims, (8) description of data collection procedure, (9) recruitment data provided, (10) justification for analytic method selected, (11) whether the method of analysis was appropriate to answer the research aims, (12) evidence that the research stakeholder have been considered in research design or conduct, and (13) strengths and limitations critically discussed [71]. In total, 2 reviewers (SF and LA) conducted quality assessments, and any discrepancies were brought to a third reviewer (AD) for additional feedback to reach a consensus. Also, these ratings were presented in a table to aid in contextualizing the narrative summary.

### Data Synthesis

A summary of findings was provided in narrative and tabular format (Multimedia Appendices 2-4) based on the outcomes reported in the studies reviewed, along with an indication of the quality of the studies resulting from our use of the QuADS tool. Since the methods used to assess university campus food environments are considerably heterogeneous, we will not perform a meta-analysis of outcomes associated with the assessments.

### Software Used

Paper screening was completed using Covidence [70]. Data extraction and synthesis were conducted using Google Sheets.

## Results

The research protocol was registered with PROSPERO (CRD42023398073) on February 8, 2023. As per the protocol, the initial queries resulting in 4502 total records have been screened by 2 reviewers (SF and LA), with conflicts resolved by a third reviewer (AD). Data extraction and analysis were completed by January 2024. This protocol will lead to a systematic review of findings that offer evidence about existing measurements and opportunities for assessing college food environments. The results may provide researchers and academic institutions with a report of best practices for examining the healthfulness of the organizational food environment. Potential implications include informing policy and programs to improve the overall campus food environment. The results have been disseminated through a peer-reviewed publication in 2024 [72].

## Discussion

### Principal Findings

The university campus food environment has been identified as a contributing factor to the emergence of an obesogenic generation [17,26]. Existing research on university food environments has focused on describing their healthfulness or evaluating population interventions aimed at improving college students’ food choices and dietary behaviors [26,27,73,74]. This study is unique in that it will provide a comprehensive picture of tools that have been used in assessing food environments where emerging adults, specifically college students, spend a significant part of their young adult lives. Compared with other review papers that broadly assess the college food environment to understand its implications for young adults’ individual dietary behaviors and risk for chronic health outcomes, this study would provide a more nuanced understanding of the tools used for such assessments at an organizational level.

This systematic review will have many significant strengths. First, it will provide a broad spectrum of methodologies for assessing the healthfulness of university food environments, including validated and nonvalidated instruments within and outside the United States. Second, we partnered with an experienced university librarian (RH) to develop a comprehensive search strategy of peer-reviewed and gray literature to identify studies that meet our inclusion criteria. Third, the study also considered all relevant study designs; qualitative and quantitative designs were considered in the inclusion criteria. Studies from different countries and regions were included to provide a global perspective of methods used for university food environment assessments. Fourth, 2 reviewers (SF and LA) were used for every stage of the systematic review, from title screening to data extraction. A third reviewer (AD) resolved conflicts as the review progressed. Finally, the study was registered with PROSPERO and followed the PRISMA protocol for systematic reviews [68].

Despite the many strengths highlighted above, an obvious limitation of the study is the use of an established time frame of publication dates as an inclusion criterion; only papers published between 2012 and 2022 were selected for the data extraction. As a result, papers that have not yet been indexed at the time of our search or more recent papers published with unique methodologies may be excluded. Further, the initial search results indicated that most of the studies included in the systematic review were conducted in developing countries. Thus, the generalizability of the findings may be limited globally.

### Conclusions

This review will contribute helpful information to address variation in practice and improve our understanding of how researchers evaluate the campus food environment designed to influence the dietary behaviors of emerging adults. As a result, this systematic review will be informative for a broader and more nuanced understanding of these terms with implications for practice and policy. Finally, we plan to provide wide-ranging consequences or recommendations that would benefit practitioners and researchers in nutrition policy design and evaluation. Notably, there is an opportunity to digitize objective campus food environment assessments to improve the data collection procedures and expand efforts to collect and compare data across institutions [75]. While this review focuses on assessing the food environment offerings, there is an opportunity to develop digital approaches to capture subjective perceptions of college students and food hall patrons [76]. Overall, this systematic review will provide a comprehensive and updated body of evidence that will contribute to designing, implementing, and evaluating interventions to improve the nutritional choices and behaviors of emerging adults, specifically college students.

## Appendix

Multimedia Appendix 1 Search strategy for the databases included in the review.

Multimedia Appendix 2 Data extraction for descriptive statistics of articles included in the study.

Multimedia Appendix 3 Data extraction for methods or tools used for the assessment of university food environments.

Multimedia Appendix 4 Data extraction for healthfulness definitions or benchmarks used in included studies.

## Abbreviations

CINAHL: Cumulative Index to Nursing & Allied Health
DOI: digital object identifier
PRISMA: Preferred Reporting Items for Systematic Reviews and Meta-Analysis
PRISMA-P: Preferred Reporting Items for Systematic Review and Meta-Analysis Protocols
PROSPERO: International Prospective Register of Systematic Reviews
QuADS: Quality Assessment for Diverse Studies
SNAP: Supplemental Nutrition Assistance Program
USDA: United States Department of Agriculture

## Data Availability

Data sharing is not applicable to this article as no data sets were generated or analyzed during this study.
