# Supplementary material for: University Food Environment Assessment Methods and Their Implications: Protocol for a Systematic Review
Source: JMIR Res Protoc. 2024 Aug 23;13:e54955. doi: 10.2196/54955 (PMC11380064; doi:10.2196/54955)
Supplement: Multimedia Appendix 1 [file resprot_v13i1e54955_app1.docx]

**Multimedia Appendix 1.** Search strategy for the databases included in the review.

| **Number** | **Database** | **Search terms** | **Number of records**  **(As of November, 2022)** |
| --- | --- | --- | --- |
| #1 | **PubMed & Cochrane Central** | “eating habit” or “eating habits” or “eating behavior” or “eating behaviors” or “eating behaviour” or “eating behaviours” or “purchasing behavior” or “purchasing behaviors” or “purchasing behaviour” or “purchasing behaviours” or “food choice” or “food choices” or “meal behavior” or “meal behaviors” or “meal behaviour” or “meal behaviours” or Food Preferences [MeSH]  AND “Food environment” or “food environments” or “calorie posting” or “calorie postings” or “caloric posting” or “caloric postings” or “calorie information” or “caloric information” or “nutritional information” or “nutrition information” or “beverage tax” or “beverage taxes” or “point of purchase item” or “point of purchase items” or “meal plan” or “meal plans” or Nutrition Policy [MeSH] or “food pantry” or “food pantries” or “food access” or “food accessibility” or dining  AND Universities [MeSH] or college* or post secondary or post-secondary or postsecondary or universit* | 1999 |
| #2 | **Web of Science & ProQuest Nursing and Allied Health** | “Eating Habit*” or “eating behav*” or “purchasing behav*” or “food choice*” or “meal behav*” or “food preference*”  AND  “food environment*” or “calor* posting*” or “calor* information” or “nutrition* information” or “beverage tax*” or “point of purchase item*” or “meal plan*” or “nutrition* polic*” or “food pantr*” or “food access*” or “food preference*” or dining  AND  universit* or colleg* or post-secondary or postsecondary or post secondary | 392 |
| #3 | **ProQuest Nursing and Allied Health** | Abstract(“Eating Habit*” or “eating behav*” or “purchasing behav*” or “food choice*” or “meal behav*” or “food preference*”) or title (“Eating Habit*” or “eating behav*” or “purchasing behav*” or “food choice*” or “meal behav*” or “food preference*”) AND abstract (“food environment*” or “calor* posting*” or “calor* information” or “nutrition* information” or “beverage tax*” or “point of purchase item*” or “meal plan*” or “nutrition* polic*” or “food pantr*” or “food access*” or “food preference*” or dining) or title (“food environment*” or “calor* posting*” or “calor* information” or “nutrition* information” or “beverage tax*” or “point of purchase item*” or “meal plan*” or “nutrition* polic*” or “food pantr*” or “food access*” or “food preference*” or dining) AND  abstract (universit* or colleg* or post-secondary or postsecondary or post secondary ) or title (universit* or colleg* or post-secondary or postsecondary or post secondary) | 125 |
| #4 | **APA PsycInfo** | DE “Eating Behavior+” or “eating Habit*” or “eating behav*” or “purchasing behav*” or “food choice*” or “meal behav*” or DE “Food preferences” or “food preference*” AND “food environment*” or “calor* posting*” or “calor* information” or “nutrition* information” or “beverage tax*” or “point of purchase item*” or “meal plan*” or “nutrition* polic*” or “food pantr*” or “food access*” or dining  DE “Colleges+” or college* or universit* or post-secondary or postsecondary or post secondary | 1239 |
| #5 | **CINAHL Complete** | MH “Eating Behavior+” or MH “Food Habits” or “eating behav*” or “eating habit*” or “purchasing behav*” or ME “Food Preferences” or “food preference*” or “food choice*” or “meal behav*” AND  “Food environment*” or “calor* posting*” or “calor* information” or “nutrition* information” or “beverage tax* or “point of purchase item*” or “meal plan*” or MH “Nutrition Policy+” or “nutrition* polic*” or “food pantr*” or “food access*” or dining ANDMH “Colleges and universities+” or college* or universit* or postsecondary or post-secondary or post secondary | 202 |
| #6 | **Google Scholar** | "campus food environment" and "assessment" AND  "university food environment" and "assessment" AND  "college food environment" and "assessment" AND  "assessment" and "college" and "food environment" | 545 (as of July 6, 2023) |
| Total | #1 AND #2 AND #3 AND #4 AND #5 AND #6 |  | **4502** |
