# Supplementary material for: University Food Environment Assessment Methods and Their Implications: Protocol for a Systematic Review
Source: JMIR Res Protoc. 2024 Aug 23;13:e54955. doi: 10.2196/54955 (PMC11380064; doi:10.2196/54955)
Supplement: Multimedia Appendix 2 [file resprot_v13i1e54955_app2.docx]

**Multimedia Appendix 2.** Data extraction for descriptive statistics of articles included in the study.

| **Assessment Description:** | | | | | | **Assessment Focus:** | | | |
| --- | --- | --- | --- | --- | --- | --- | --- | --- | --- |
| **Author, Year** | **QuADS Score** | **Country** | **Institution Description** | **Assessment Type** | **Assessment Scope** | **Campus Food Environment (Individual level only)** | **Eateries** | **Vending** | **Convenience or Food Stores** |
|  |  |  |  |  |  |  |  |  |  |
