# Supplementary material for: University Food Environment Assessment Methods and Their Implications: Protocol for a Systematic Review
Source: JMIR Res Protoc. 2024 Aug 23;13:e54955. doi: 10.2196/54955 (PMC11380064; doi:10.2196/54955)
Supplement: Multimedia Appendix 3 [file resprot_v13i1e54955_app3.docx]

**Multimedia Appendix 3.** Data extraction for methods or tools used for the assessment of university food environments.

| **Author, Year** | **Tool Description** | **Assessment Methods** | **Primary Findings Regarding Food Environment** |
| --- | --- | --- | --- |
|  |  |  |  |
