# Supplementary material for: University Food Environment Assessment Methods and Their Implications: Protocol for a Systematic Review
Source: JMIR Res Protoc. 2024 Aug 23;13:e54955. doi: 10.2196/54955 (PMC11380064; doi:10.2196/54955)
Supplement: Multimedia Appendix 4 [file resprot_v13i1e54955_app4.docx]

| **Healthy Definition** | **Articles** |
| --- | --- |
|  |  |
